# Patient safety incidents associated with EMR use: Results of a national survey of Swiss physicians

**DOI:** 10.1177/20552076251403204

**Published:** 2026-01-21

**Authors:** David Schwappach, Wolf Hautz, Gert Krummrey, Yvonne Pfeiffer, Raj Ratwani

**Affiliations:** 1 Institute of Social and Preventive Medicine (ISPM), 27210 University Bern, Bern, Switzerland; 2 Department of Emergency Medicine, Inselspital University Hospital, 27210 University of Bern, Bern, Switzerland; 3 Institute for Medical Informatics (I4MI), Bern University of Applied Sciences (BFH), Biel/Bienne, Switzerland; 4 Harmfree Healthcare, Wädenswil, Switzerland; 5 Institute of Nursing Science (INS), 89389 Faculty of Medicine, University of Basel, Basel, Switzerland; 6 2613 MedStar Health National Center for Human Factors in Healthcare and Georgetown University School of Medicine, Washington, DC, USA

**Keywords:** Electronic medical record, usability, patient safety, incident reporting, survey

## Abstract

**Objectives:**

Electronic medical records (EMRs) are increasingly recognized as a contributing factor to patient safety incidents. Clinicians’ experiences can reveal EMR-related risks that may otherwise go unnoticed. This study explores EMR-related patient safety incidents reported by physicians across diverse care settings, institutions, and EMR products.

**Methods:**

A national sample of Swiss physicians was surveyed online and asked whether they had experienced a patient safety incident related to EMR use within the previous four weeks. Free-text descriptions of incidents were analyzed thematically using a structured, multi-step procedure.

**Results:**

Of the 1933 inpatient and outpatient physicians who completed the survey, 23.9% (*n* = 398) reported experiencing an EMR-related safety incident in the previous four weeks. Half of these incidents (49.7%) had not been formally reported (e.g. through critical incident reporting or IT channels). A total of 385 incident descriptions were analyzed, revealing seven emergent themes: (1) patient identification and selection errors (16.7%), (2) system reliability and performance issues (15.8%), (3) interoperability and system integration (8.8%), (4) usability, interface, and design problems (21.8%), (5) system errors and unexpected behavior (8.8%), (6) security and access control (2.6%), and (7) problems with order entry, decision support, alerting, and verification (25.2%). There were considerable differences in the patterns of events reported in relation to the used EMR system.

**Conclusions:**

Physicians reported a broad range of EMR-related safety problems, particularly related to ordering functionalities and usability, many of which were not formally recorded. In addition to broader socio-technical strategies, such as user training, incident reporting, and alignment with clinical workflows, systematically incorporating clinicians’ experiences into EMR design is required to guide advancements in patient safety.

## Introduction

Electronic medical records (EMRs) are the most widely used health information technology in many healthcare systems of high-income countries. Physicians now spend a significant amount of their time working with computers, making the EMR a central working tool.^1^ EMRs are also increasingly used to connect physicians across care settings, for example, hospitals and outpatient physicians, or offer an interface for patients to access their health-related data, for example, test results. The EMR's role in organizing, ordering, delivering, and documenting care is fundamental. The adoption of EMRs has contributed to improvements in patient safety. Notably, electronic medication systems have been associated with reductions in prescribing errors, although high-level evidence remains mixed.^2,3^ Higher scores on the Leapfrog computerized physician order entry evaluation tool have been linked to lower incidences of preventable drug events.^4^ However, substantial variation persists in the safety performance of contemporary EMR systems.^5^ This variability is influenced both by the design and usability of the EMR itself and by organizational factors, such as implementation strategies and continuous improvement initiatives.^6–8^ Indeed, clinicians’ subjective perceptions of EMR usability are strongly correlated with EMR safety performance, highlighting the importance of considering end-user perspectives for targeting safety improvements.^9^

Despite their huge potential for safer care, however, EMRs have also been identified as contributors to delayed care and patient safety incidents, both in hospital and outpatient care, and have been linked to patient harm and death.^10,11^ In their analysis of incident reports from general practitioners, Magrabi et al. identified problems that had previously existed with paper records but became more pronounced with IT use (e.g. multitasking between multiple open patient records), but also safety issues unique to digital systems.^12^ These included challenges with user interfaces that could hinder safe navigation, as well as errors occurring during the migration of clinical records from one system to another. Ratwani et al. studied 9000 patient safety incident reports related to EMR and medication issues in pediatric care.^13^ In a third of the incidents, usability problems were identified as contributing to the medication event. In a national study, Palojoki et al. analyzed 2379 EMR-related patient safety incidents reported by more than 20 hospitals over a two-year period in Finland^14^: Nearly three quarters of all reports (73%) related to problems in human–computer interaction.

While prior studies provide valuable insights into EMR-related safety issues, reliance on formal incident reporting systems introduces several challenges that may obscure the true scope and variety of risks. First, patient safety incidents capture only a small fraction of the total safety issues that actually occur in care settings.^15^ Second, EMR-related safety incidents are not always attributed to the EMR system and may be categorized as medication events or in other categories that are more apparent to the reporter. For example, a medication error due to poor EMR design, such as poor visual display and system feedback problems, is likely to be categorized as a medication issue rather than being recognized as EMR-related issue.^13^ Finally, safety issues related to EMR use are rarely reported transparently, and many are likely not reported at all. One important barrier to transparent reporting is the presence of “gag clauses” in contracts between vendors and healthcare providers, which restrict clinicians from sharing information, for example, screenshots, about safety issues they encounter.^16–18^ Thus, knowledge about specific risks remains limited.^19^ Hence, supplemental sources for identifying EMR related safety issues are needed.

Previous studies have analyzed incident reports to identify EMR-related safety issues, often focusing on specific aspects such as usability or medication processes,^2^ or specific areas of healthcare, such as hospitals or outpatient settings. However, less is known about the broader spectrum of EMR-related incidents as experienced by physicians in daily clinical practice. To close this gap, we conducted a nationwide survey among physicians to explore patient safety incidents associated with EMR use across diverse settings, institutions, and EMR products. Examining clinicians’ real-life experiences across different healthcare contexts within one country can provide valuable insights into EMR-related risks that might otherwise remain unrecognized or underreported.

## Methods

This analysis is based on data obtained in a national study on EMR usability. The quantitative results of the study have been reported elsewhere.^20^

### Survey

As part of the larger cross-sectional Swiss EMR usability survey, participants were asked whether they experienced any patient safety incident related to EMR use: “*Please think about the last four weeks: Was there any incident while working in ‘MySystem’ that, in your view, posed a risk to patient safety?*.” (“MySystem” was replaced by the name of the EMR system the participant selected from a list of EMRs.) We did not provide a formal definition of “patient safety incident” as the survey was designed to capture physicians’ own perceptions of EMR-related risks. The term “incident” is commonly used in the patient safety field and is generally familiar to physicians. The wording was intentionally broad to include both events that reached the patient and led to patient harm as well as near misses, acknowledging that at the point of reporting it is often unclear for the involved clinician whether harm actually occurred. If yes, they were asked open-ended questions to provide details about the incident and whether they had reported the incident to any instance (supervisor, colleagues, critical incident reporting system, hospital IT or EMR vendor). Several personal and work-related characteristics were also assessed to identify whether the respondents worked in ambulatory or hospital care and in what role they used which kind of information system.

### Participants

Members of the Swiss Association of Resident and Attending Physicians, a voluntary organization representing physicians across all regions and healthcare settings, were invited to participate (*n* = 20,245). The online survey^21^ was distributed in December 2024 via email with individual access codes. Non-respondents received two reminders. The study was exempt from review by the lead ethics committee of the Canton of Bern (Req-2024-01052). Participants were informed about the voluntary nature of survey participation in the invitation email. At the beginning of the survey, they received detailed information about the study. Continuing with the survey was considered as providing informed consent to participate, in accordance with the protocol approved by the ethics committee.

### Data analysis

We used descriptive statistics to report responses as frequencies. Chi-square tests were conducted to examine associations between respondents’ characteristics and incident reporting. The free-text incident reports were thematically analyzed in several steps. First, the data were anonymized by a researcher to ensure confidentiality, for example, names of persons, hospitals and vendors were substituted with letters. Second, the reports were coded with the support of ChatGPT, which was used as a research collaborator. ChatGPT was prompted to iteratively develop a coding scheme inductively from the data, without deleting, selecting, or changing any content^22,23^ (see Appendix). A human researcher provided feedback on random samples of 20 comments suggesting revisions to the coding scheme and ChatGPT then applied the refined coding scheme to the complete dataset. Prompting was conducted on 17 February 2025 using the web interface of GPT-4o under a ChatGPT “plus” license. Third, the coded output was validated by human researchers through direct comparison with the original data to ensure that no changes, deletions, or additions occurred. Importantly, ChatGPT's role was limited strictly to the coding stage. The subsequent thematic analysis, identifying overarching, emergent themes, was performed entirely by a human researcher. Here, emergent themes refer to patterns and concepts that became apparent through iterative examination of the data, rather than being predefined. This approach enabled the identification of recurring issues and underlying meanings not evident from individual reports alone. Finally, a researcher not involved in prior steps of data analysis applied the final theme classification to a 15% random sample of comments. Chi-square tests were used to evaluate whether the distribution of incident types differed significantly across reporting channels or EMR products.

## Results

Of the 1438 hospital physicians and 495 ambulatory care physicians completing the survey, 23.9% (*n* = 398) declared to have experienced an EMR-related safety incidents in the past four weeks. Table 1 reports respondents’ characteristics. Compared to the complete sample, participants reporting an incident were younger (48.1% vs 35.9% under 35 years, *p* < 0.001), more likely to work in a hospital (90.7% vs 68.8%, *p* < 0.001), and to be a resident physician (45.6% vs 32.4%, *p* < 0.001). The medical specialties general internal medicine (42.7% vs 36.4%), anesthesiology (10.6% vs 5.1%), and intensive care (6.5% vs 2.2%) were overrepresented among those who reported an incident. The hours of EMR training received were not associated with reporting an incident (*p* = 0.054). However, those with <12 months experience with the current EMR were more likely to report an incident (42.6% vs 27.3%, *p* < 0.001). Participants indicated they had informed their colleagues (41.0%) or supervisors (34.7%) about the incident, followed by reporting to a critical incident reporting system (32.2%) and IT department or vendor (25.6%). A substantial proportion (49.7%) chose not to report the incident through formal channels (reporting system or IT), although many shared it informally with colleagues or supervisors. Notably, 17.6% did not report the incident to anyone. A total of 372 respondents (93.5%) provided details of incidents which were included in the thematic analysis. As some participants reported multiple, independent events, the total number analyzed was 409. Some events (*n* = 24) could not be classified because the descriptions lacked sufficient detail to determine the potential contribution of the EMR. For instance, a report only stating “medication administration error” does not specify whether the error was related to EMR functionality (e.g. order entry, documentation, alerting) or to other factors.

**Table 1.** Characteristics of survey participants who reported at least one EMR-related patient safety incident in the previous four weeks.

	*n* (%)
*N*	398
Age	
<35 years	187 (48.1%)
35–44 years	101 (26.0%)
45–54 years	72 (18.5%)
55–64 years	25 (6.4%)
>64 years	4 (1.0%)
Gender	
Female	220 (56.7%)
Male	168 (43.3%)
Medical specialty	
General internal medicine	170 (42.7%)
Anesthesiology	42 (10.6%)
Surgery	19 (4.8%)
Gynecology and obstetrics	7 (1.8%)
Intensive care medicine	26 (6.5%)
Cardiology	4 (1.0%)
Pediatrics and adolescent medicine	27 (6.8%)
Medical oncology	6 (1.5%)
Nephrology	8 (2.0%)
Neurology	12 (3.0%)
Orthopedic surgery	11 (2.8%)
Psychiatry and psychotherapy	10 (2.5%)
Rheumatology	3 (0.8%)
Urology	5 (1.3%)
Other^a^	48 (12.1%)
Work setting	
Medical practice	37 (9.3%)
Hospital	361 (90.7%)
Medical role	
Medical student	1 (0.3%)
Resident	181 (45.6%)
Senior physician	89 (22.4%)
Consultant	20 (5.0%)
Hospital specialist	18 (4.5%)
Lead physician	48 (12.1%)
Chief physician	15 (3.8%)
Visiting consultant	10 (2.5%)
Other^a^	15 (3.8%)
Type of hospital	
University hospital	106 (29.5%)
Cantonal hospital	96 (26.7%)
Regional hospital	104 (29.0%)
Psychiatric clinic	13 (3.6%)
Rehabilitation clinic	8 (2.2%)
Other specialty clinic	32 (8.9%)
Hospital department	
Anesthesiology	40 (11.2%)
Surgery, incl. subspecialties	42 (11.7%)
Gynecology and obstetrics	7 (2.0%)
Internal medicine, incl. Subspecialties	133 (37.2%)
Intensive care medicine	27 (7.5%)
Emergency department	35 (9.8%)
Pediatrics	23 (6.4%)
Psychiatry and psychotherapy	13 (3.6%)
Other^a^	38 (10.6%)
Type of practice	
Dual/group practice	37 (100.0%)

a Other summarized over detailed categories.

Seven emergent themes were identified in the data: (1) patient identification & selection errors (*n* = 65, 16.7%), (2) system reliability & performance (*n* = 61, 15.8%), (3) interoperability & system integration (*n* = 34, 8.8%), (4) usability, interface & design issues (*n* = 84, 21.8%), (5) system errors and unexpected behavior (*n* = 34, 8.8%), (6) system security & access control issues (*n* = 10, 2.6%), (7) order entry, clinical decision support, alerting and verification (*n* = 97, 25.2%). Interrater agreement between researchers in theme assignment was very good with Kappa = 0.86. Table 2 provides the thematic classification of EMR-related safety incidents with category descriptions and illustrative physician quotations.

**Table 2.** Thematic classification of EMR-related safety incidents with category descriptions and illustrative physician quotations.

Patient Identification & Selection Errors (*n* = 65): Errors related to selecting or managing the wrong patient records, leading to misdiagnoses, incorrect treatments, misplaced orders, or documentation mix-ups. Because windows do not close automatically, medication was prescribed for the wrong patient due to a lack of overview in a hectic work environment. I searched for the patient's information, clicked on their name, and then navigated to their data. However, I later realized that I had mistakenly remained on the previous patient's record instead. CT scan was ordered for the wrong patient. Opening a different patient's file when switching dossier subcategories. An operation consent form was uploaded to the wrong patient's file as a PDF in the reports section.
System Reliability & Performance (*n* = 61): System crashes, outages, slow performance, unresponsive interfaces, that disrupt clinical workflow and impact or delay patient care. The blood ordering process failed in an emergency. The poor workflow distracted focus from the patient, compromising safety. Emergency O-negative blood had to be given instead of a fully compatible transfusion. Patients for surgery (including emergency procedures) could not be loaded into the EMR. Night support was unavailable, delaying operations by up to 90 min. Complete system crash. Outpatient consultations had to proceed without records or information. The entire EMR system crashed for about an hour, blocking quick access to data. Lab tests and imaging had to be ordered manually. Changes to PRN medications are almost impossible to make, as the system crashes every time, requiring verbal orders to compensate. System crashed during anesthesia handover, leading to loss of critical patient information.
Interoperability & System integration (*n* = 34): Lack of integration and synchronization between devices or systems, across departments or hospitals, requiring manual data entry or duplicate documentation, leading to missing or conflicting patient information and fragmentation. At hospital X, it is sometimes impossible to retrieve diagnoses from other internal clinics. This nearly led to a contraindicated medication being prescribed. Data from the nighttime POCT lab was not automatically transferred. Two systems (EMR 1 and EMR 2) are used simultaneously, but they are not linked. This means that if I have Patient A open in one system, it is not necessarily the same patient in the other system, increasing the risk of errors. Incorrect medication transfer after an internal patient transfer. The interface between the emergency department and the intensive care unit (ICU) does not work: ICU staff must manually enter printed EMR records, and emergency department medications must be dictated instead of electronically transferred.
Usability, Interface & Design Issues (*n* = 84): Poor system usability, confusing layouts, hidden or unclear information, or counterintuitive navigation that cause errors, or lack of oversight in medical management or increased cognitive burden on healthcare providers. Sub-diagnoses are only displayed when clicked on. Allergy information is poorly structured: The top-right section lists general allergies (e.g. nuts, fruits), but only when clicked do drug allergies (e.g. Co-Amoxicillin) become visible. Lab values cannot be compared because they rarely align at the same level. Constantly searching for lab values, which often appear in different locations, makes oversight and comparison extremely difficult and time-consuming. The medication view for physicians differs from that for nurses, leading to confusion. When senior physicians correct a final discharge report, the corrections are not reflected in the problem list. Since the problem list is referenced for future admissions, this leads to a loss of important clinical information. Information is rigidly displayed from 7 AM to an unspecified hour. While prescribing a medication, I overlooked that it had already been prescribed at 6:50 AM, leading to an overdose.
System errors and unexpected behavior (*n* = 34): Bugs, errors, or unintended system changes. Unexpected system actions and automation without user control causing unintended consequences. Change of medication times by the system when the date changes (administration is automatically set to the current time after a medication was rescheduled from morning to today). Incorrect dispensing unit set as the default, for example, Fragmin in pieces instead of IU. If the nursing staff measures a diabetic patient's blood sugar at a non-standard time, the system still suggests the meal-time insulin dose. A steroid was prescribed for the following day with a new dosage, and the previous order was stopped. However, the system unexpectedly changed the start date to “immediately,” leading to a double dose. The hospital's in-house Ibuprofen (400 mg) dosage was not pre-configured in the EMR, leading to an automatic adjustment to 800 mg.
System Security & Access Control Issues (*n* = 10): Unauthorized access, blocked user actions, role mix-ups, or authentication flaws, system lockouts, or security vulnerabilities that compromise patient data integrity or restrict necessary clinical actions or delay care. Users make changes or orders under another person's account (whether nurse or doctor). The (wrong) prescription was made by a physician who was not involved in the patient's care and was working in another hospital. Browser-based system: Users log in under another identity if they open a URL link from another user.
Order entry, clinical decision support, alerting and verification (*n* = 97): Issues in prescribing, scheduling, modifying, or discontinuing medications or interventions (e.g. imaging, lab). Lack of clinical guidance, including incorrect dosages, duplicate prescriptions, or unintended discontinuations. Missing, unclear, or excessive alerts (allergies, interactions, overdoses), contributing to alert fatigue or unnoticed risks. Verification issues, refer to mismatches or gaps in system-supported checks at the point of order processing (e.g. failure to flag duplicate orders, missing dose-range checks, or absence of alerts for contraindications). Duplicate medication orders were not recognized by the system. Conversely, the system flagged non-duplicate medications as duplicates (e.g. different opioid medications prescribed in different treatment phases such as post-anesthesia recovery vs inpatient care). The system allows dangerously high doses to be prescribed for neonates through the standard interface. I reactivated a paused Hydromorphone prescription that had mistakenly been set at 2 mg IV by a junior colleague. A warning alert would have been very helpful. The EMR does not calculate total fluid intake from medications, infusions, and nutrition. When IV medications are changed, this can lead to significant alterations in fluid balance. No alert for anticoagulation with NOACs, which led to two lumbar punctures. Dosage error, mix-up between mg and ml (Oxynorm). The patient had not yet received the dose.

Consequences for the involved patients were sometimes explicitly described and ranged from none to very serious, including death. In a considerable number of reports, the emotional language indicated the physicians’ shock and sense of helplessness in response to the EMR malfunction (“I simply couldn’t find the information in the system – it was a nightmare.” “You have the patient in the ED urgently needing care and five people stare at the screen and wait minutes for the system to load the patient record”). In some reports, physicians indicated not feeling heard by hospital IT, EMR vendor, or hospital leadership when informing about safety-related problems with the EMR (“Unfortunately, at the hospital, a culture of silence prevents any criticism of the EMR. Speaking out is discouraged or even actively suppressed.”; “The issue was reported to Critical Incident Reporting System and to EMR support, but no response or improvement followed.”).

**Figure 1.**
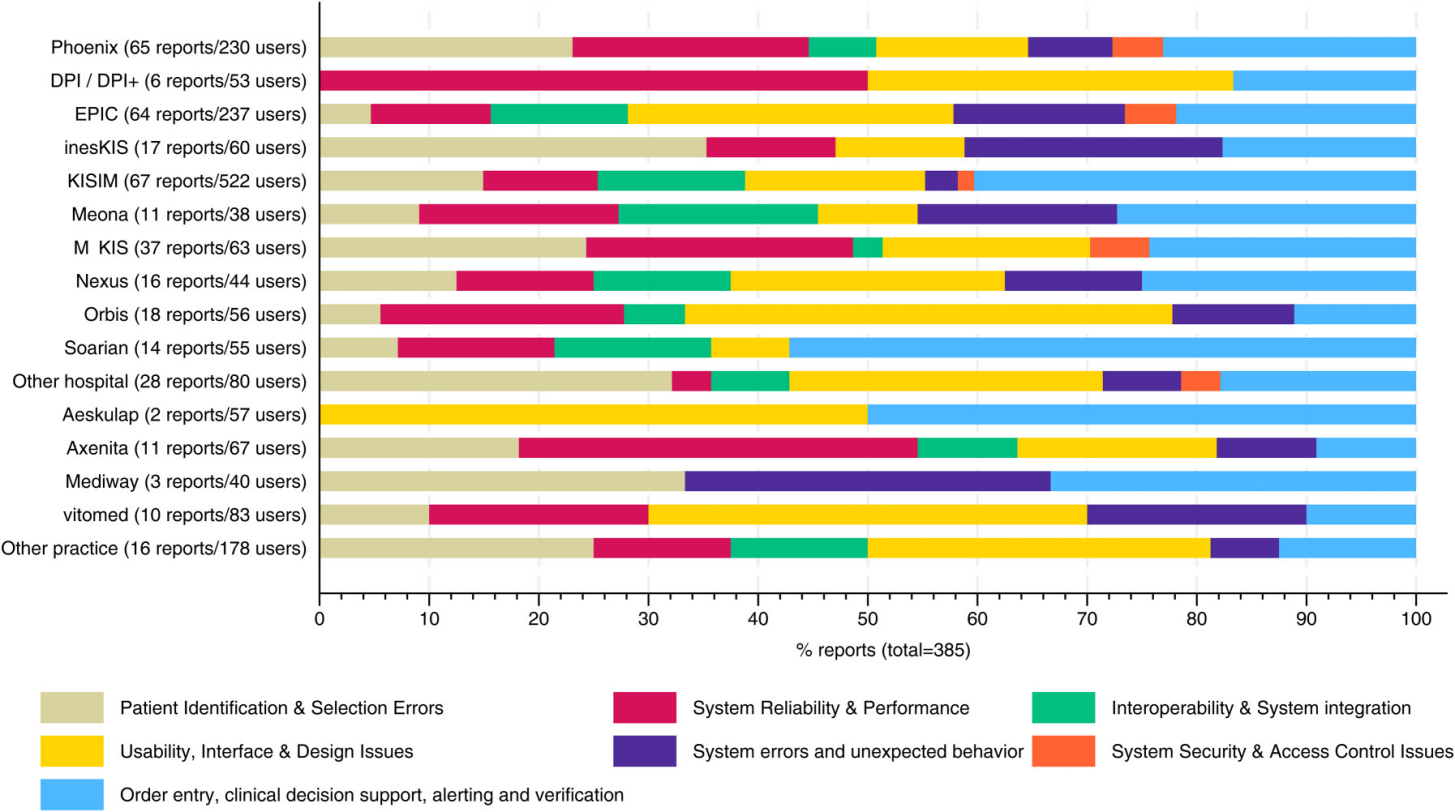
Patient safety incidents reported by type of incident and used EMR system. EMR labels indicate the number of users in the sample and the number of incident reports submitted by these users.

There was a significant association between the type of patient safety incident and whether it was reported through formal channels (incident reporting system or IT department) or informal channels (colleagues or supervisors) (*p* < 0.001). Incidents related to system reliability and security were most frequently reported through formal channels, with rates of 70.5% and 70.0%, respectively. By contrast, formal reporting was much less common for usability-related incidents (29.7%) and patient identification errors (36.9%), highlighting that these types of incidents are often shared informally. For incidents involving order entry and alerting, 45.4% were reported through formal mechanisms. There were considerable differences in the patterns of incidents reported across EMR systems (Figure 1). The association between incident type and EMR product was statistically significant (*p* = 0.017). For example, patient identification and selection errors were more frequently reported by users of EMR Phoenix (23.1%) than by users of EMR Epic (4.7%), despite a similar number of users in the sample. Likewise, system reliability and performance problems were reported more often by Phoenix users (21.5%) compared to Epic users (10.9%). In contrast, usability issues were more frequently reported by Epic users (29.7%) than by Phoenix users (13.4%).One-third of all incidents related to order entry, clinical decision support, alerting, and verification were reported by users of EMR Kisim. In this group, the category also represented the largest share of all incidents (40.3%). Interoperability and system integration issues were relatively rarely reported by users of EMR M-Kis. System security and access control problems were reported exclusively by hospital physicians. Among outpatient physicians, usability issues were the most frequently reported incidents, followed by patient identification errors and system reliability problems.

## Discussion

To the authors’ knowledge, this study provides one of the first overviews of EMR-related patient safety incidents based on a national survey of physicians employed across different healthcare settings and using various EMR products. Nearly a quarter of physicians reported experiencing a patient safety incident related to EMR use within the past four weeks, with some incidents resulting in significant patient harm, as assessed by the reporting physician. The events reported cover a broad range of problems with a concentration on ordering functionalities and usability. Many incidents involve the interplay between the EMR system and end-user factors, such as workload, multitasking, or environmental pressures. While our analysis focuses on EMR-related risks, these events reflect the broader context in which clinicians interact with the system.

This finding underlines the importance of usability testing and user-centered design of EMR functionalities. Indeed, there is growing evidence that user-centered (re-)design of clinical processes in the EMR can help prevent errors. For example, Orenstein et al. investigated how user-centered design of pediatric blood product orders influence pediatric transfusion ordering practices and blood product-related safety events.^24^ Though safety outcomes did not change in the study period, they found significant decreases in ordering errors, for example, transfusions administered faster than 5 mL/kg/h. Mazur et al. examined the impact of a usability modification in EMR test result organization, demonstrating both decreased cognitive workload and improved performance in managing abnormal test results.^25^

Compared to safety incidents reported to national reporting agencies, we observed both similarities and notable differences. In line with the findings of Howe et al., incidents related to ordering, alerting, system automation and defaults, as well as interoperability, were common in both studies.^2^ However, because our survey focused exclusively on physicians and not nurses, medication administration errors – which were frequently reported in the Howe et al. study – were rarely reported by our sample. In contrast, Howe et al. limited their analysis to usability issues, which explains the absence of system downtimes, slow performance, poor technical reliability, and access control issues in their findings. These issues were, however, prevalent in the reports we analyzed. This observation is consistent with prior findings from a voluntary incident reporting database in one Australian state,^26^ where 24% of computer-related patient safety incidents were attributed to general technical problems rather than to human–computer interaction issues. Thus, while usability problems represent a significant share of contributing factors in safety incidents, technical issues such as system downtimes and poor reliability frequently led to potentially harmful delays in care and should not be neglected.

Our results allow comparison of the reported incidents across EMR systems. However, several study design weaknesses need to be considered. The number of incidents reported and the representation of problem types are influenced by voluntary reporting and recall. Problems directly experienced by physicians during safety-critical clinical tasks are more likely to be reported. Indeed, our results indicate that physicians may experience strong emotional responses to EMR problems that hinder them from providing safe care, for example in emergencies. Such experiences are likely to affect the memory and reporting of incidents. In addition, differences in user experience, training, or local reporting practices may also shape what is reported. Thus, the raw number of incidents should only be cautiously compared between different EMR systems. Nevertheless, differences in the patterns of problems may provide insights into the relative strengths and weaknesses of the systems.

For example, in our data, some EMRs were more prone to patient misidentification and performance problems, as reported by end-users, while others were more susceptible to usability and interoperability issues. Our data also suggests that a shorter duration of working with a particular EMR system is associated with increased incident reporting. Limited individual EMR experience typically occurs when physicians change work environments or when a system is newly implemented in a hospital. The observed association could be explained either by true, higher rates of incidents in the early periods of adoption, for example, caused by poor system performance, lack of user experience, or insufficient end-user training prior to EMR functionality implementation, or by a higher willingness of clinicians to report events in the early phases of implementation, or both. Initial rising rates of observed (rather than reported) errors and adverse events and poor perceived usability have been described shortly after implementation of new EMR systems.^2,27^ It thus seems plausible that our results do not only reflect reporting effects.

Physicians in our study preferred to inform their colleagues and supervisors over hospital IT and formal incident reporting channels. It is concerning that many incidents will thus not only be unavailable for improvement or mitigation strategies in the affected health care organizations but will also be missing from large-scale risk assessments. For example, many studies rely on patient safety event reporting systems or requests filed as IT help desk tickets as sources to understand patient safety threats associated with EMR use.^28^ In an analysis of the National Reporting and Learning System in England and Wales over a period of 12 years, only 0.00019% of all incidents reported related to failures in health IT.^29^ Our results indicate that these data sources are lacking a substantial – and maybe specific – proportion of IT-related safety incidents leading to an underestimation of the associated harm. There are a few ways to address this. One way is to make it easier for physicians to report by enabling a reporting feature directly in the EMR. If designed and implemented rigorously, the reporting physician could include a screenshot and other metadata that would better describe the EMR safety issue and provide the necessary context and audit trail to address the issue. Certainly, health information technology risk assessment should therefore include additional methods, such as surveys, simulations or direct observation, to get a broad overview.^8,30,31^

This study has certain limitations. First, the survey response rate was relatively low, which is not unusual for recruitment strategies relying on mailings among members of professional associations. Physicians who chose to participate may differ systematically from non-respondents, for example in their interest in patient safety or digitalization. Second, as outlined above, physicians may not be able to recall all the safety issues they faced in the last four weeks resulting in under reporting. In addition, physicians may not feel comfortable describing these issues which would also lead to underreporting. Third, the level of detail provided by reporting physicians varied considerably across reports. For example, some reports included information on the consequences of the incident for the patient, whereas in others it remained unclear whether any harm had reached the patient. A stratification of incidents by risk or severity was not feasible, as the incident reports did not consistently provide sufficient detail to allow for reliable assessment. Moreover, within each incident category both low- and high-severity events were present, making categorization by risk level impossible. Finally, we do not have a way to verify that the reported issue actually is an EMR-related safety issue.

## Conclusions

Several participants in our survey reported that even serious event reports or requests for assistance directed to their IT departments received no response, and some felt discouraged from openly discussing their EMR-related concerns. Given that patient safety today can be significantly affected – either enhanced or compromised – by health information technology, effective collaboration (including training) among clinical users, healthcare IT departments, and vendors is crucial. By surveying physicians through their professional organization and ensuring anonymity with respect to their employers, we likely increased participants’ willingness to report incidents. Nevertheless, meaningful improvements in patient safety require that clinicians’ experiences and concerns are actively acknowledged by those responsible for EMR design. To achieve this, a collaborative model of improvement should be established that brings together frontline EMR users, industry, and healthcare system leadership so that these safety issues can be addressed in a timely collaborative fashion.

## Supplemental Material

sj-docx-1-dhj-10.1177_20552076251403204 - Supplemental material for Patient safety incidents associated with EMR use: Results of a national survey of Swiss physicians Supplemental material, sj-docx-1-dhj-10.1177_20552076251403204 for Patient safety incidents associated with EMR use: Results of a national survey of Swiss physicians by David Schwappach, Wolf Hautz, Gert Krummrey, Yvonne Pfeiffer and Raj Ratwani in DIGITAL HEALTH
