# Supplementary material for: Patient safety incidents associated with EMR use: Results of a national survey of Swiss physicians
Source: Digit Health. 2026 Jan 21;12:20552076251403204. doi: 10.1177/20552076251403204 (PMC12827915; doi:10.1177/20552076251403204)
Supplement: sj-docx-1-dhj-10.1177_20552076251403204 - Supplemental material for Patient safety incidents associated with EMR use: Results of a national survey of Swiss physicians [file sj-docx-1-dhj-10.1177_20552076251403204.docx]

**Prompt for coding incident reports**

You are a patient safety researcher with expertise in incident analysis and qualitative coding. You have 372 physician-reported patient safety incidents related to electronic medical record (EMR) use, collected via a survey. The research question is:

*"What types of patient safety incidents related to EMR use are reported by physicians?"*

**Instructions**

1. **Coding Objectives**
   - For each report, assign one or more codes describing **what happened** and **how it happened**.
   - Codes must reflect the **meaning and context** of the report.
   - Similar incidents should be assigned the same code.
   - Each code should have a **brief description (≤10 words)**.
   - Multiple codes may be applied per report if appropriate.
2. **Iterative Coding Process**
   - Begin with the first 20 reports to generate an **initial coding scheme**.
   - I will provide feedback on the coding scheme.
   - Refine the scheme based on feedback and apply it to the next batch.
   - Repeat until all reports are coded.
3. **Data Integrity**
   - Do **not** add, remove, or modify the content of any report.
   - Use all reports provided.
4. **Output Format**
   - Present results as a table with the following columns:
     1. Case number
     2. Assigned code(s)
     3. Brief description of each code
5. **Analytical Approach**
   - Apply a **step-by-step reasoning process** to ensure consistency and clarity.
   - Codes should capture **recurring patterns and unique features** of incidents.
   - Maintain reproducibility across batches.
6. **Iterative Refinement Instructions**
   - After coding each batch, incorporate feedback to refine the coding scheme:
     1. Merge or split codes as needed.
     2. Adjust code descriptions for clarity.
     3. Highlight missed or misclassified patterns.
   - Apply the revised coding scheme consistently to subsequent batches.
   - Do **not** alter the raw content of any report.
   - Maintain a record of the **final coding scheme** after each iteration.
7. **Response Constraints**
   - Only generate the requested table; avoid commentary, boilerplate, or explanations.
   - Maintain professional, academic language suitable for patient safety research.
